# Pathways and identity: toward qualitative research careers in child and adolescent psychiatry

**DOI:** 10.1186/s13034-024-00738-8

**Published:** 2024-04-29

**Authors:** Andrés Martin, Madeline DiGiovanni, Amber Acquaye, Matthew Ponticiello, Débora Tseng Chou, Emilio Abelama Neto, Alexandre Michel, Jordan Sibeoni, Marie-Aude Piot, Michel Spodenkiewicz, Laelia Benoit

**Affiliations:** 1 Child Study Center, Yale School of Medicine, New Haven, CT USA; 2 QUALab, Qualitative and Mixed Methods Lab, a collaboration between the Yale Child Study Center, 230 South Frontage Road, New Haven, CT 06520 USA; 3 CESP, The Centre de Recherche en Épidémiologie et Santé des Populations, Paris, France; 4 Departamento de Psiquiatria da Faculdade de Medicina da, Universidade de São Paulo, São Paulo, Brazil; 5 Inserm U1018, CESP, Team DevPsy, Paris-Saclay University, Villejuif, France; 6 Department of Psychiatry, McGill University Faculty of Medicine, Montréal, QC Canada

## Abstract

**Objective:**

Qualitative research methods are based on the analysis of words rather than numbers; they encourage self-reflection on the investigator’s part; they are attuned to social interaction and nuance; and they incorporate their subjects’ thoughts and feelings as primary sources. Despite appearing well suited for research in child and adolescent psychiatry (CAP), qualitative methods have had relatively minor uptake in the discipline. We conducted a qualitative study of CAPs involved in qualitative research to learn about these investigators’ lived experiences, and to identify modifiable factors to promote qualitative methods within the field of youth mental health.

**Methods:**

We conducted individual, semi-structured 1-h long interviews through Zoom. Using purposive sample, we selected 23 participants drawn from the US (n = 12) and from France (n = 11), and equally divided in each country across seniority level. All participants were current or aspiring CAPs and had published at least one peer-reviewed qualitative article. Ten participants were women (44%). We recorded all interviews digitally and transcribed them for analysis. We coded the transcripts according to the principles of thematic analysis and approached data analysis, interpretation, and conceptualization informed by an interpersonal phenomenological analysis (IPA) framework.

**Results:**

Through iterative thematic analysis we developed a conceptual model consisting of three domains: (1) *Becoming* a qualitativist: embracing a different way of knowing (in turn divided into the three themes of priming factors/personal fit; discovering qualitative research; and transitioning in); (2) *Being* a qualitativist: immersing oneself in a different kind of research (in turn divided into quality: doing qualitative research well; and community: mentors, mentees, and teams); and (3) *Nurturing*: toward a higher quality future in CAP (in turn divided into current state of qualitative methods in CAP; and advocating for qualitative methods in CAP). For each domain, we go on to propose specific strategies to enhance entry into qualitative careers and research in CAP: (1) *Becoming:* personalizing the investigator’s research focus; balancing inward and outward views; and leveraging practical advantages; (2) *Being:* seeking epistemological flexibility; moving beyond bibliometrics; and the potential and risks of mixing methods; and (3) *Nurturing*: invigorating a quality pipeline; and building communities.

**Conclusions:**

We have identified factors that can support or impede entry into qualitative research among CAPs. Based on these modifiable findings, we propose possible solutions to enhance entry into qualitative methods in CAP (*pathways*), and to foster longer-term commitment to this type of research (*identity*).

**Supplementary Information:**

The online version contains supplementary material available at 10.1186/s13034-024-00738-8.

…we must reckon that numbers can say only so much, and that we need to better listen and better represent the voices of those under our care, especially of those who have been unheard or disenfranchised for far too long. We believe that less quantity and more quality can help us meet those aspirations [1], p.3.

Qualitative methods of research favor the analysis of words over that of numbers, which are in turn the main focus of quantitative approaches. With its preference for thoughts, ideas, feelings, and other aspects of internal life, qualitative inquiry is particularly well suited for psychiatry [2]. Moreover, with child and adolescent psychiatry’s (CAP’s) interest in exploring the interactions between groups of individuals and their role as interconnected social actors, qualitative methods are especially well suited for the discipline. The link between CAP as a subject matter and qualitative methods as a favored research approach would appear to be a natural one.

If only it were. Qualitative studies in CAP are in fact scant. For example, a search for the terms “qualitative” AND “child OR adolescent” AND “psychiatry” using Google Scholar (date of access: December 1, 2023) returned 2,588 entries, in contrast to a comparable search yielding 75,196 entries when substituting the first term with “quantitative,” representing a 29-fold difference favoring quantitative over qualitative publications. Stratifying the same analysis across decades reveals a more telling pattern: the fraction of qualitative studies from among all those published in CAP during the decade ending in 2013 was 2% (359/18,154); by the following decade, the proportion had doubled, to 4% (2,229/54,454). The number of qualitative studies increased sixfold from one decade to the next, compared to a threefold change for quantitative studies. In short, these trends reflect how even if the absolute number of qualitative studies in CAP has remained low, there has been a proportional increment in their publication, reflecting growing interest in qualitative methods in CAP research.

Glancing at the table of contents of scholarly outlets suggests yet another story. Specifically, it is not uncommon for leading CAP journals to publish no qualitative studies for years on end—when at all. Qualitative science often finds its way into different periodicals, some without any mental health focus. The point here is how spliced qualitative science remains from the more “mainstream” science and publications of CAP, which remain almost exclusively focused on quantitative methods [3]. Conferring a “second class science” status to qualitative methods [4] has implications not just for scholarship, but for patient care, including to “contribute voice to advance equity in health [5].” CAP has been slow in the uptake of qualitative methods seen in other specialties (including oncology, primary care, and medical education), in which there has been a movement toward a more collaborative person-centered approach, one with more consideration for the lived experience of patients and their caregivers [6].

Partly at the root of this tension is what has been termed *epistemological unconsciousness* or *positivist orthodoxy* [7], a worldview prevalent in medicine and the sciences that has a built-in preference for objectivist (i.e., quantitative) rather than constructivist (i.e., qualitative) views. The rise of the evidence-based movement, which continues to prioritize quantitative research, has introduced further challenges to the qualitative community [8]. Moreover, the relative scarcity of qualitative studies in CAP may not be entirely coincidental. Falissard et al. [1] have posited three likely contributors. First, a focus on children, the research agenda of whom is commonly overtaken by that of adults. Despite higher returns on earlier life stage investments, decisions on funding allocations—from education to healthcare to research—rarely prioritize children. Considered through the lens of *childism* [9], the systematic societal prejudice against children, CAP research priorities are commonly overshadowed by those of general psychiatry and medicine more broadly, much as qualitative methods can become lost under a quantitative hegemon.

Second, the focus of CAP on mental health has become almost interchangeable with a focus on brain disease and the particular tools for its “proper” study: genetics, brain imaging, clinical trials, and other “objective” instruments. Despite the advances in these areas, laboratory tools cannot access important aspects of mental health function, such as mind and relationships. The “decade of the brain” has left limited room for the mind, and in so doing, contributed to reifying the “brainlessness and mindlessness” that Leon Eisenberg warned against in the late 1980s [10]. A disillusionment with biomedicine and its tools has introduced an epistemological malaise into medicine, which those working under a qualitative framework are striving to address.

Finally, qualitative research’s connection to psychoanalysis may have proved a burden to its application in psychiatry, particularly CAP. With its interest in words, thoughts, feelings, and deep reflection, psychoanalysis would appear a natural precursor to qualitative methods. Psychoanalytic literature can have an uncanny resemblance to qualitative papers, such as biographical or narrative studies. Despite the similarities and shared roots in sociology, anthropology, and literature, qualitative methods in mental health research have suffered under the shortcomings of analysis, including its insularity and exclusive focus on the individual. In the end, two disciplines rooted in interpretivism drifted apart; the methodological shortcomings [11] and stigma of psychoanalysis cast a shadow on the promise of the fledgling qualitative field.

Provocative as these hypotheses are, they are speculative and not based on actual data. To our knowledge, there are no studies that have empirically investigated the reasons propelling or impeding research careers in qualitative methods, and certainly none in the field of CAP. Faced with this gap in the literature, and through what may be considered a "meta" approach, we used *qualitative methods* to interview CAPs actively involved in different stages of *qualitative research*. The overall goal of our effort was to identify factors, particularly modifiable ones, that could enhance the number and methodological rigor of this type of research in CAP, and to help enrich the pipeline of future investigators dedicated to the intersection of the two disciplines: in short, to help grow quality CAP research and those dedicated to it.

## Methods

### Participants and individual interviews

We conducted individual, semi-structured interviews organized around a guide consisting of 23 sensitizing questions (Additional file 1: Appendix S1). Each of the interviews was 1 h long and conducted thorough videoconferencing using Zoom (San Jose, CA). Semi-structured interviewing is a flexible, commonly used method in qualitative research in healthcare that uses a prepared list of questions to guide researchers and participants to “co-create meaning” through an exploration of thoughts, feelings, and opinions, especially those around potentially sensitive or personal topics [12, 13]. Interviewees were not necessarily asked all of the sensitizing questions. Using convenience and purposive sampling [14], we selected 23 participants drawn from the US (n = 12) and from France (n = 11), and equally divided in each country across seniority level. All participants had published at least one peer-reviewed qualitative article, and were classified as junior if having 5 or fewer years of post-doctoral experience; those with 6 or more years were considered senior. Ten participants were women (44%).

### Data collection, qualitative analysis, theoretical framework, and reflexivity

We recorded all interviews digitally and transcribed them for analysis using Deepgram (deepgram.com, San Francisco, CA). We uploaded the collection of transcripts into software for qualitative analysis (NVivo version 12; QSR International, Melbourne, Australia). We coded the transcripts according to the principles of thematic analysis, a qualitative approach involving the active construction of overarching patterns and meaning across a dataset [15, 16]. Thematic analysis allows the flexible and atheoretical exploration of rich text data to construct themes that “reframe, reinterpret, and/or connect elements of the data” without developing a final theory. We approached analysis, interpretation, and conceptualization informed by interpretative phenomenological analysis (IPA), an approach based in psychology that attends to participants’ inner realities [17, 18]. We adopted an inductive approach, with our research questions evolving beyond the initial sensitizing questions.

Three authors coded independently throughout the study span in an iterative manner, with coding and interviewing interdigitated to allow for the inductive approach to inform subsequent interviews. They then combined and triangulated codes, and established the final codebook to eliminate redundancies, clarify domains, themes, and subthemes. In this way we ensured theoretical sufficiency [19]. Each final code was supported by quotes from more than one participant.

All authors were interviewed and took part as study participants; five authors served as interviewers, and four as data coders. We were attentive to positionality, with no instance of a hierarchical or working relationship between interviewers and interviewees. The closeness between all authors/study participants, the fact that as CAPs they were looking into their own guild and in some way into themselves, and the subject matter of the study itself, all required careful attention to everyone’s reflexivity [20] during the discussion and write-up process. The dual role as researchers and participants provided broader insights, as the resonance between the views of non-researcher-participants and those of researcher-participants improved the triangulation and comprehension of the gained insights [21]. This view is aligned with the “patient-as-partner” approach to health care, education and research, in which participants are not limited to just providing feedback on results. Instead, they are included as participants from the very beginning of the conceptualization of the project and until its end, e.g., the in-depth review of its ensuing manuscript [22]. Through involvement not only as investigators but as beneficiaries, stakeholders, and participants, our group effort exemplified the principles of Participatory Action Research (PAR) [23, 24].

### Ethics approval

We obtained ethics approval from the Yale Human Investigation Committee (Protocol # 2000035118), which considered the study exempt under 45CFR46.104 (2) (ii). We informed participants about the goals and methods of the study and provided a copy of the consent form. The form noted that participation was entirely voluntary and optional; it described in detail the study procedures and potential risks, including discomfort during the interview and a small risk of loss of confidentiality, minimized by encryption consistent with institutional policies. We recorded each participant’s consent before starting their interview. In writing our findings, we adhered to best practices in qualitative research, as articulated in the COREQ guidelines [25].

## Results

**Fig. 1:**
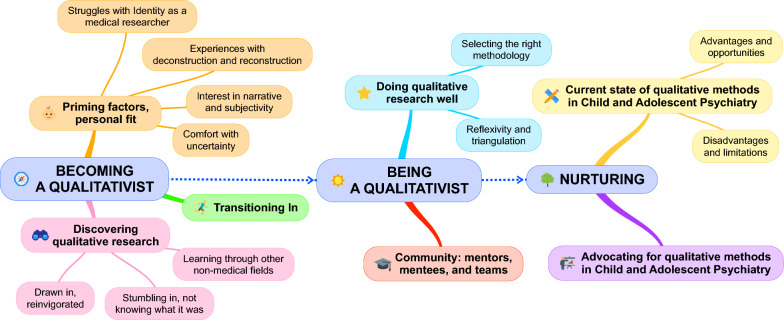
Concept map: domains and themes toward qualitative research careers in child and adolescent psychiatry

Through iterative thematic analysis we developed a conceptual model, depicted in Fig. 1, consisting of three domains: (1) *Becoming* a qualitativist: embracing a different way of knowing; (2) *Being* a qualitativist: immersing oneself in a different kind of research; and (3) *Nurturing:* toward a higher quality future in CAP. We go on to describe each domain in the three subsections and corresponding tables that follow. We organized the tables following a similar rubric: (a) definition of each domain; (b) division into underlying themes and subthemes; and (c) support of constructs through representative quotations.

### *Becoming* a qualitativist: embracing a different way of knowing

#### Priming factors, personal fit

We identified four commonalities in personal and professional characteristics among most study participants. Specifically, shared traits included ways of approaching scientific inquiry and knowledge creation, as well as struggle and unease with prevailing medical models of research (Table 1).

**Table 1:** *Becoming* a qualitativist: embracing a different way of knowing

Theme	Subtheme	Representative quotation
1.1. Priming factors, personal fit	1.1.1. Comfort with uncertainty	I'm excited about research that involves applying theory to think about how the social world shapes psychiatric illness. The social sciences intersecting with medicine give me hope for a better understanding of the political and social determinants of health, of so much in our field that is imprecise and uncertain
	1.1.2. Interest in narrative and subjectivity	My roots are very much in the arts and the humanities, and it doesn’t feel that I need to put them on hold for the sake of research. To the contrary, they enrich the connections and creativity of my work
	1.1.3. Experiences with deconstruction and reconstruction	Research that is based on concrete, almost cinematographic, practical stories; on disjointed scenes from everyday life that are somehow stitched together; that is rooted in the experiential as the starting point
	1.1.4. Struggles with identity as a medical researcher	For me, research used to be something deserted, where everything was reduced and abstracted, and then the world was summed up with a few major trends, but that were far removed from reality
1.2. Discovering qualitative research	1.2.1. Learning through other, non-medical fields	He was almost saying, “I’m sorry to tell you, but I think you are just as interested in sociology as in medicine, and I think qualitative methods may be a good fit for you.” I was intrigued, as all the questions I had were always related to how people behave with each other
	1.2.2. Stumbling in, not knowing what it was	What's interesting is that it feels like a very old love. As if I knew it all along. I was surprised at first when I learned that there was such a thing. But once I started learning what this thing was about, it didn’t seem so new, it rather seemed a new application of what I had been doing all my life, which is reading and making sense of books and people and their life stories
	1.2.3. Drawn in, recalibrated, reinvented	I started my academic career very late because I didn’t think I was cut out for it at all, I didn’t think I had the skills, I didn’t see myself doing quantitative research. I had the maturity to handle it, but not the background, the thinking, the network. Qualitative provided a less daunting entry point; it was welcoming and made clinical sense: it rang true
1.3. Transitioning in		My quantitative work has enriched my qualitative one and vice versa. There is a point in trying to diversify a research portfolio. Two points, actually: a scientific one and a funding one

##### Comfort with uncertainty

Whether stating it explicitly or not, virtually all participants demonstrated an ability or interest to navigate gray zones of uncertainty. They embraced a “less rigid form of creativity,” one in which their mental pliability and freedom to be playful in solving problems were valued skills:

I found that qualitative research both fosters and demands a certain level of intellectual flexibility, like mental gymnastics, which quantitative research is not as conducive to. *(French female, FF)*

Participants reported feeling more at ease with relative, rather than purportedly absolute truths. They valued scientists who pursued “indeterminate spaces,” seen as role models who favored salient questions over predetermined methods: “those who go to where the science needs to go.” Participants did not decry qualitative methods and their underlying philosophy (positivism), so much as thrive under the opportunities afforded by a complementary approach (qualitative) and philosophy (constructivism).

Our societies are not only modern societies. They are also postmodern societies. And with postmodernity you are allowed to consider that universalism does not exist. Singularity exists. Identities exist. *(French male, FM)*

Whether established or fledgling, this group of qualitativists welcomed opportunities to change their minds and reevaluate intellectual preconceptions. They sought ways to complement their uncertain understanding of reality by entering the “narrative truths” of their subjects.

##### Interest in narrative and subjectivity

With qualitative methods being as reliant on words as they are, it is only natural that the approach exerts a strong pull on physicians and scientists who are drawn to the humanities, literature in particular. A plurality of participants described qualitative methods using terms not common in the sciences: “applied literature,” “humanities-adjacent,” “bridging medicine and science with humanities and art.” One participant described the qualitative interview and subsequent analysis as “capturing, curating, and sharing life stories; as if reading, rather than writing, life experiences needing to be shared.” *(American male, AM).*

Some participants experienced traditional medical research as too confining and prescriptive of what it valued as “real (i.e., objective.)” They felt limited by an approach that was too simplistic, linear, basic, and formulaic for their sensibilities. By contrast, in qualitative methods, they found a venue to explore political and social determinants of health more adequately, to think about philosophical principles and their applications. Participants considered their own views, however biased they may be, as also relevant and informative, partly because as full participants in the research endeavor, they saw the interdependence between the perceiver and the perceived, the revealing exchange between themselves and their subjects, for

as Merleau-Ponty puts it, in *“The Flesh of the Real”:* I discovered the world from the fact that I myself am in it. *(FM).*

From early on in their experience, several participants described a natural inclination to “think about thinking” and an appreciation for the relativity of psychological truths. For them, the qualitative approach was more than a methodology; it proved a veritable way of approaching and making sense of the world, of moving between “reality” and overarching philosophical conceptualizations:

What I liked about qualitative research was that it was a way to think about philosophical ideas from an imaginative and interpretive angle–but always based on real-life data. *(American female, AF)*

For a minority of participants, the road to qualitative methods could be considered escapist: less a pull towards it than a push away from quantitative approaches. For some of them, a dislike of lab work, a sense of overly delayed gratification, or a feeling of abstraction to the point of irrelevance were considerable motivators away from quantitative methods. In one or two instances, the avoidance of “real, i.e., quantitative” research fortuitously led them to qualitative research, which they perceived as “more human, more nuanced, more forgiving, and more welcoming and indeed encouraging of subjective experiences.” *(FF).*

##### Experiences with deconstruction and reconstruction

Qualitative methods are inherently deconstructive: by exploring the connections and assumptions between text and meaning, particularly by attending to the inner workings of language. Transcripts—fragments of a life transcribed—are the most common building block for the deconstruction performed by qualitative studies. More precisely, qualitative research is an intellectual endeavor of construction and deconstruction:

It’s like Penelope waiting for Ulysses: weave, reweave, unweave...it doesn’t matter that it’s iterative or that there are changes as you go along. In fact, that’s precisely where the action is. *(FM)*

Among over half of participants, it was not just texts that were being deconstructed: They thought of their own formative experiences source material for deconstruction. This was particularly the case for those participants who experienced themselves as “outsiders,” whether through immigration, language or culture, social disconnection, or distance from the house of medicine, “as if fighting for the legitimacy of our field.” *(AM)* These members saw a link between their personal experiences reconciling dualities with their academic draw to qualitative methods, to sense-making work. Qualitative deconstruction and reconstruction proved a way of putting to use their experiences navigating more than one world at once, of unlocking realities taken for granted by others. The joys of qualitative research were at times described as a paradigm shift that allowed for the “unlocking” of new and singular realities:

Quantitative methods by their very nature simplify data; every time you take an analytic step in order to find commonalities, you lose something about the subjects as individuals. In qualitative, by contrast, you highlight their uniqueness, you bring them into sharper relief. *(AF)*

##### Struggles with identity as a medical researcher

Throughout professional development, many participants had struggled to reconcile their medical or clinical selves with their research interests. After negative experiences or feeling disconnected from projects, several had gone on to avoid or become uninterested in traditional medical research. When first exposed to qualitative methods, several participants were surprised: of their existence, their relevance, and their role within medicine, where “I didn’t know that I, as an MD, was allowed in; it was something for social scientists, not for me.” *(FF).*

Research had meant statistics. During this time, no one ever told me there was an alternative approach. I was already reading sociology, philosophy, social science, but more as something that I was interested in than as something you could use in medical research. This is how they would do research in the humanities, good for them, but this was not research for physicians. *(FF)*

Quantitative methods were described by some as distant, manipulated, or divorced from real meaning; as dry and reductionistic. Perhaps more than anything, participants sensed a tension between patient-proximal vs patient-distal approaches, admiring the ability of qualitative methods to access and reveal people’s lives through the specific details and richness of shared stories. Early experiences often took novice researchers aback, with “the granularity of the qualitative stories being so intense that you cannot but pay attention.” *(AM)* One participant described relief on learning there was a place for their preferred aspects of research, as “qualitative is like a place where valuable and meaningful things end, things that otherwise would not have gone anywhere else.” Placing similar sentiments into a broader context of medicine within society, another participant described hope in a renewed sense of patient-centered care, one in keeping with qualitative approaches:

As we find that medicine gets more impersonal, cold, and distant, and that the doctor doesn’t know their patient, maybe that's part of the re-invigoration of qualitative approaches: To say that as physicians we care, we really care about story. About *your* story. *(AF)*

It is worth noting that over half of participants had conducted and published mixed-methods research, yet only one or two mentioned the approach during their interviews, perhaps reflecting an internalized dichotomy of two methods often seen at odds with each other. And yet, this quote captured a sentiment that would likely be endorsed by most subjects:

I think that there is a role for both. There’s a role for numerical research and I still do some of it and, you know, it's great but it’s different. And I have a very small list of mixed methods papers, and I would like over time to have more of those, because I think that when you bring the two approaches together, the science is particularly rich. *(FM)*

#### Discovering qualitative research

Participants’ first acquaintance with qualitative methods was often indirect, unexpected, or serendipitous: an encounter by virtue of relevant non-medical experiences. Several participants found themselves in qualitative territory only once immersed in a field they hadn’t realized they had fully entered. Once into qualitative methodology, several described experiences of rejuvenation, recalibration, or veritable professional reinvention.

##### Learning through other, non-medical fields

Most study participants reported having very limited exposure to qualitative methods or research during medical school. As such, their entry into the field was only rarely influenced by having been exposed to seminal qualitative papers or influential talks. Instead, common pathways in were through personal experiences in psychotherapy, or by a rekindling of early experiences with disciplines “outside” of medicine: anthropology, sociology, literature, or occasionally through “medicine-humanities” hybrid disciplines like medical education, history of medicine, or global health.

Interest in psychotherapy and psychoanalysis, both in theory as in personal experience, led some participants to question whether there was a potentially important crosstalk between the disciplines:

I started talking to my supervisor about philosophy of science, anthropology, and the problems I saw with psychoanalysis. I wondered if there was something good in psychoanalysis that we could salvage? That’s what we were talking about, and that’s what first led to my interest in qualitative. *(FM)*

Classic papers in the psychoanalytic literature, with their thick descriptions, held an immediate appeal, activating an entreaty to reinvigorate contemporary approaches to medical research and writing that appeared currently dry and untextured:

We've jettisoned our whole case report culture in medicine, and maybe our qualitative research now supplants or overlaps that empty space to an extent. *(AM)*

A common sentiment among many was that the biopsychosocial model of medicine had given short shrift to the social component, “the last of the three, and not by coincidence.” That unintentional omission compounded the mothballing of social science skills that participants feared during their socialization as physicians. As a result, social content and skills became relegated to atrophy and disuse. By contrast, what often first opened the doors to qualitative methods was approaching social- and language-based questions in medicine, particularly in the context of knowledgeable and supportive mentors:

He told me “You are really asking yourself sociological questions; this is really good, because you think like sociologists do.” I was not shamed nor made to feel a dilettante; I was encouraged to keep trying, and I did. *(FF)*

##### Stumbling in, not knowing what it was

For some, a serendipitous path into qualitative methods occurred through a chance encounter with a mentor or research team, or through peers who identified interests and skills well suited to the methodological approach. A few others, focused and self-driven, found the path intentionally. For yet others, qualitative was more of an incidental find than an active search. “Someone names it for you. Meeting an informant or guide (a friend, a colleague), who unexpectedly tells you about qualitative research—and that you may already be in the midst of it. Unsuspecting you”:

It's almost like in Molière’s *Le Bourgeois Gentilhomme*, in which Monsieur Jourdain speaks in prose without knowing it’s prose. This is a little bit of what I experienced: “You’re already doing it, but don’t know that you’re doing it.” *(FM)*

##### Drawn in, reinvigorated

No matter how one first enters the qualitative field, novices soon find a qualitativist peer—someone with similar ways of thinking about clinical phenomena and research. It is a discovery of a team, as much as of a method; of a social as much as of a scientific direction. People get enmeshed through the encouragement and referrals of others, and qualitative methods are social in a unique way: the research cannot be done in isolation, as.

Two people cannot triangulate, certainly not one person: you need three. That summarizes for me the inherent team-based nature of qualitative work. Being invited into a team got me started. I have not looked back since. *(AF)*

About half of the senior participants described arriving late in their professional careers to qualitative methods. For all of them, the new approach, the new colleagues, the new of seeing their work, was described in terms such as “reinvigoration,” “recalibration,” “renovation,” “reinvention,” or “getting me out of a career hole.” A few without prior qualitative background described the turning point as “nothing short of a mid-career renaissance.” Two participants used the word “love” in describing the experience:

I don’t regret anything. It would have been very nice to discover this back in medical school, but I eventually discovered it and it’s fine. I’ve had a very interesting, fun career, and in some ways I’m glad that I'm finding it late on because now I have like this brand new love affair with medicine. *(AM)*

#### Transitioning in

A common sentiment on entering the field was around stigma, the notion that qualitative research was somehow “less than,” that it could perhaps even hurt career prospects. This internalized bias could manifest as fear of irrelevance, of engaging in lower-quality research perhaps not worth doing, of being relegated to lower-impact journals. Some described worries about their efforts being irrelevant or self-indulgent. For those who found a home in qualitative work, its meaningfulness outweighed those considerations, even if deemed “risky” to their training or career prospects:

It's awful to say this, but there’s a certain utilitarianism, certain things that you need to do to progress in the academic ladder. Traditional approaches were okay only for a while, before I saw that those questions were not important enough to keep me going. *(FM)*

On finding a group of peers invested in qualitative methods, the challenge moved away from devaluation into misrepresentation: how to make others understand the role and the value of as different a way of approaching medical research. How to defend the approach confidently to others, to uphold its legitimacy, and to explain its basic tenets. One participant put it in gendered terms:

“There seems to be a gendered aspect to the type of research: qualitative research (soft/feminine) not considered as legitimate as quantitative (hard/male). It is a jaundiced view, of course, but may explain a skew to women participating in qualitative, as if there was some kind of feminization of empathy.” *(AM)*

A gradual settling in followed for most, through the external validation from mentors, peers, and role models, and after presenting and publishing their work. These externalities led to internal acceptance, to letting go of what had been expected as necessary to conduct “real” research (one participant stated how they had been socialized to believe how “if you don’t suffer, it’s not real research”). *(FF)* The more comfortable in their new qualitative selves, the more insecurities were dropped, and the focus shifted to meaning rather than external standards.

The tension between qualitative and quantitative approaches was certainly not a Manichean one. Several participants spoke of the power of mixing the two approaches; of how questions could be enhanced by combining the approaches into mixed methods designs. For some, the advantages proved not only scientific, but fiscal as well: Funding agencies looked favorably upon (when not outright expecting) a qualitative component to otherwise traditional grants. But in the final analysis, personal meaning was of utmost importance:

I'm just trying to do something that’s important and meaningful to me. I'm not going to commit to a research career that is merely strategic: “If I do x, then I'll get funding y; rinse and repeat...” I would be betraying myself. For the past ten years, I’ve become consistent with my inner compass. Qualitative research has been central to that. *(AF)*

### *Being* a qualititavist: immersing oneself into a different kind of research

#### Quality: doing qualitative research well

Once into the fold of qualitative methodologies and research, participants reflected on two broad areas of importance: 1) Selecting particular methodologies most suitable to their background, personality, and specific research questions; and 2) Finding and fostering a community of peers committed to qualitative work (Table 2).

**Table 2:** *Being* a qualitativist: immersing oneself into a different kind of research

Theme	Subtheme	Representative quotation
2.1. Quality: doing qualitative research well	2.1.1. Selecting the right methodology	The amount of data can be daunting. And you learn real quick that software can’t do it for you. You have to actually read through everything, to think through everything, to make sense of it yourself
	2.1.2. Reflexivity and triangulation	Well conducted qualitative research is a highly accomplished effort at decentering, of making one’s own subjectivity explicit in order to make room for the subjectivity of others
2.2. Community: mentors, mentees, and teams		I need to find the right distance. I do pretty well on my own—up to a certain point. So, the idea is to be available without being too present either. Except that when I give the signal, when I ring the bell, it's because I'm really not coping, I need help, I need to be reframed

##### Selecting the right methodology

In settling on the right types of methodologies for their work, participants described three general stages they had experienced or seen in others.

The first was common at the outset: learning by doing rather than through explicit teaching; practice over theory, as through an emphasis on coding. “Learning by doing” is what pulled in many novices at the outset, the ability to start coding and analyzing from the get-go, without lengthy prerequisites necessary. In this early phase of exhilarated discovery, software and small samples could make the work appear deceptively simple. The use of software could result in a pause in critical thinking, a sense that “coding is like coloring by numbers,” of simply sorting and filing away. However initially attractive, this phase represented a shortcut that participants had to unlearn with experience as their coding became more nuanced. Small samples were also appealing, both in the efficiency of data collection and in their make-up: children, families, patients, human lives in their full richness. Although these subjects were often the very reason that made qualitative methods of interest in the first place, the richness of qualitative samples could lead to getting lost in details and hyper-specificities, especially when considering the large amount of data created during qualitative analysis.

A qualitative study could theoretically require one person’s story, because one person is an entire universe within themselves. One person being enough: so much richness in the singular. *(AM)*

A second stage indicated growing knowledge about and ease with specific methods. For some, it required “unlearning traditional quantitative approaches,” although most participants recognized that their original skills, including around scientific rigor, collaboration, and experience with scholarly writing, transferred and generalized. Turning from quantitative to qualitative (and particularly mixed) methods was described more as supplementing and enriching research skills than as supplanting an original skillset with a newer one. Learning to do qualitative research well and gaining comfort with its specific methods reinforced interest and eased joining a new community and way of thinking. The transition had its challenges, as when navigating the degree of structure vs freedom in research, and of dealing with “hard” vs “soft” findings.

It's nice that there aren’t specific statistical tests that you have to apply based on a given dataset. No correct answers. Instead, there are many correct ways. It’s nice to have that freedom. Freeing, but also terrifying. *(AF)*

In selecting the methodologies of their choice, some participants identified a cautionary tale in the “overdoing” they had seen in other colleagues, even in themselves. In such instances, methodological fetishization could prevail over pragmatism and derail original goals. An over-emphasis on the right methodology could result in a tree-for-forest problem, a focus away from a project’s central question.

If I could use my magic wand, I would make a qualitative world where there are fewer “methodological churches,” but rather different ways to work, a respect for how we work with the same finality of helping kids. And in that world, every clinician would be a little bit of a researcher, and every researcher would be a little bit of a clinician, or at least know what the other one does, and take interest. We must fix this if we want to consolidate the field. *(FM)*

##### Reflexivity and triangulation

Reflexivity can be conceptualized as the awareness and incorporation of the role of oneself in a research project. It is a central feature of qualitative research, and one that distinguishes it from the “selfless” and “ego-neutral” tenets of its quantitative counterpart. Through reflexivity, personal thoughts, feelings, past history, even prejudices become a filter through which to see and conceptualize research findings; in quantitative methods, these same elements would be dismissed as bias. As a result, qualitativists engage in interpretative, reflective work as a matter of course. The ensuing challenges include being able to balance the “dose of oneself,” of being deeply reflective and attuned to one’s role in the project, while remaining able to go against personal prejudices and beliefs, of remaining humble by “being able to think against yourself.” Psychiatrists and mental health professionals may be particularly attuned to this approach: On the one hand, with few procedures or objective laboratory findings to rely on, we often practice with little more than ourselves as the tool of diagnosis and healing; on the other, our work is largely one of reflection, of thinking about others, about understanding another’s subjectivity through our own, of doing so while not centering the experience on ourselves.

The qualitative approach makes it possible to think about both the object perceived and the object that perceives it and tries to explain the structures of perception and how they can influence the result of this perception. In other words, an approach that aims to make subjectivity explicit, to make explicit the consciousness that perceives and assumes it, and to put it to work. *(FF)*

A cautionary note regarding over-thinking in qualitative work is warranted: Participants warned that the potential to over-interpret and pathologize can be a barrier. Psychoanalytic thinking in particular can be as helpful as it can lead astray. Reading too much theory or searching for the perfect theoretical framework can prevent the researcher from listening to the participants’ experience. Psychologizing can be a challenge for early qualitativists, “a shortcut blunting their own formulations and creativity.”

I used to “psychologize” much more. And progressively, I found a way not to fall into this psychologization or sociologization trap and remain at an experiential level and at a phenomenological descriptive level. But I think it wasn’t easy, and it’s very tempting at the beginning to also put your clinical intuition, and defense mechanisms and all of this, and it's only with time that you can clean that and really respect the qualitative methodology as it should be. *(FM)*

For many participants, triangulation offers the remedy for potential solipsistic thinking. Triangulation is a principal tenet of qualitative rigor, requiring confirmation between at least three sources (e.g., coders, texts, timepoints) before accepting a proposed finding. By comparing codes in an iterative fashion, and by casting light on the interview, coding, analysis, and writing stages, investigators can retain the transparency of their findings. They can also hold personal views and biases in check. Similar to clinical work, the subjective “truth” is approached only by crossing diverse subjective experiences. Qualitative work cannot be done in a social vacuum: Qualitative work is necessarily teamwork.

We already have a dimension of mutual surveillance. I don’t mean that in a bad way, but in a good way, i.e., we watch each other’s work. And that’s why I think this research is so enriching. *(AF)*

#### Community: mentors, mentees, and teams

Mentorship, supervision, and guidance were among the most fulfilling aspects of entering qualitative research—and among the most frustrating. In terms of frustration, some experienced abandonment early on, as when mentors encouraged participants to write a manuscript but then failed to follow through in email exchanges, or when participants were blindly congratulated for great teamwork despite feeling lost in a project. Some mentors had felt let down or used by students who upon graduation vanished and abandoned a paper without publishing it. In the list of discontents, there was a common theme agreed on by all: the shortage of highly skilled and knowledgeable mentors with a proven track record. Development could be stunted by the lack of senior experts available. Navigating qualitative methods without mentors could be unfulfilling, unsatisfying, even lead to premature departure from the field. For mid-career qualitativists, a lack of senior mentors had forced some to “grow up” ahead of schedule, placing them in a vulnerable position as they tried to keep developing while also breaking down barriers, leading, and carrying others on their proverbial backs. They may not have been strong enough at that stage of their careers to carry themselves and others at once.

The problem with qualitative research is that you're not always properly supervised. I think it’s sufficiently new in psychiatric research that people with little experience are put in as trainers. When I was a young researcher, I was asked to be the advisor of a resident doing qualitative research, even though I wasn’t really ready. *(FF)*

Notwithstanding the small number of qualitative mentors available, particularly in CAP, virtually all participants had found at least once such mentor during their professional development. Meetings had been either fortuitous (as when preassigned to someone) or by active choice, often after meeting with several candidates. Those choices were based on relational fit (or in the evocative French term, by “la resonance,” or “echo”). The “echolocation” could work as well for individuals as for groups. Indeed, group-wide mentorship, typically comprising members of different backgrounds and levels of seniority, was highly valued and sought after. People are drawn to another’s work and actively create community with them; they enjoy and benefit from diversity among reviewers and their perspectives:

We've got people doing phenomenology, grounded theory, sociology…The idea is precisely to show that everyone can work together and get around the table to move a question forward, whatever the method of the paper. Which is different, I think, from the old ways of doing research, when it was really about either being with us or against us. *(FF)*

### *Nurturing:* toward a higher quality future in child and adolescent psychiatry (CAP)

#### Current state of qualitative methods in CAP

Participants envisioned a larger role for qualitative methods in the future of CAP, and for the contribution they could make in getting the fields to more frequently and more seamlessly come together (Table 3).

**Table 3:** *Nurturing:* toward a higher quality future in child and adolescent psychiatry

Theme	Subtheme	Representative quotation
3.1. Current state of qualitative methods in child and adolescent psychiatry	3.1.1. Advantages and opportunities	Zoom is free, recording is free, and now that transcribing is virtually free. It used to be that transcribing was a significant cost. And now with AI, it is good enough that it’s essentially free. So really the only cost is our time. If you try to do this with quantitative, with brain imaging, with genetics, it’s impossible. So, I'm hopeful for qualitative methods, especially in resource-limited settings
	3.1.2. Disadvantages and limitations	I’ve gotten pushback from people saying this doesn’t count as a scientific study when what they really mean to say is, “I'm not familiar with qualitative methods. I'm not fit to review this paper.”
3.2. Advocating for qualitative methods in CAP		Winnicott said it first: there is no such thing as a baby. There are no children in a vacuum, devoid of parents, families, schools, communities. As child and adolescent psychiatrists, we are family and systems doctors. Qualitative methods are perfect for our way of thinking about the world

##### Advantages and opportunities

By virtue of being so inherently part of the research—through their reflexivity, subjectivity, interpretation, or contextualization—many participants conveyed a sense of ease within the qualitative realm. From an epistemological perspective, they considered the possible research questions and the specific qualitative methods as getting closer to the truth; and if not to *the* elusive truth, at least to meaningful moments of discovery that said something new or described new phenomena.

You get to bring yourself as the investigator, you get to bring your true self. You don’t have to hide behind anything. You bring whatever, you know, blips, limitations, liabilities, blind spots, and whatever. It’s all good. *(AM)*

It helped me find an “ecological niche,” a term that I've taken from this work. A niche where I feel I want to be, where I feel like I fit and belong. And my existing skills, interests, and strengths naturally fit with it as well. *(AF)*

For some, qualitative methods permitted a view at the cracks in traditional, accepted forms of research. But rather than offering just a perch to see those limitations, it allowed entry through different ways: a range of variety, novelty, and puzzles not typically a part of medicine, and a framework through which to synthesize a range of different inputs and interests. The work led to more “existential relevance,” more resonance with personal values, to a wider lens into the world. Indeed, work with marginalized and historically excluded voices seemed especially well suited for qualitative methods, giving “the appropriate tools for this work of community epistemological justice,” all the more during this “DEI moment.”

Qualitative methods held an appeal for investigators with a more social and interpersonal approach, when interviewing different actors such as children, parents, teachers, and social workers in the case of CAP. The approach also proved a good fit for international work and collaborations, insofar as much of the work (whether international or not) had migrated to videoconferencing platforms. The low costs and high quality to interview and transcribe leveled the research playing field, making qualitative studies affordable at a distance and in resource-constrained settings. Aside from its social dimension, qualitative methods proved consistent with clinical interview skills and the refinement of reflective and empathic skills:

I sometimes try to do my clinical interviews as if they were qualitative interviews. I have learned so many things that are different from the usual stuff, from the diagnostic criteria and what not. And so, qualitative methods sanitized me to the fact that we’re far from done and have so many more things to know. *(AF)*

Participants also valued qualitative skills in answering the entreaty to be more spontaneous in our clinical actions:

Something that sticks out to me about child and adolescent psychiatry and about qualitative research is just how playful both of them are.

##### Disadvantages and limitations

Several participants described having been made to feel like second-class researchers, yet unsure how much their counterparts understood qualitative methods and their role within medicine. This sentiment was especially biting for psychiatrists, who had invariably been made to feel like “stepchildren” of medicine with a yearning to be welcome as full-fledged, legitimate doctors. One participant described the “dual stigma” compounding their identities as a CAP and as a qualitativist. Both stigmas, it needs to be said, are internalized forms of bias, the internalization of other individuals’ and communities’ negative perceptions.

A very different and very concrete disadvantage had to with funding. The ability to fund a long-term career in qualitative methods was challenging. Even if the costs are generally low, funding one’s time can be challenging, particularly through traditional federal sources. Participants shared experiences with funding through private foundations, but were generally frustrated in their attempts at larger grants. Some felt the need to include a qualitative component in proposals “as a hook to get reviewers’ attention, or to reassure them that we know how to conduct research.” Alternatively, others described a sense of “tokenization” when asked to include a (small) qualitative component as part of a larger grant.

#### Advocating for qualitative methods in CAP

Several of the characteristics of qualitative research make it particularly well fitting for early career researchers, regardless of specialty: Small samples and low budgets are usually sufficient, and projects can be completed in a relatively short period of time. In the case of child psychiatry, additional advantages include the interest in subjectivity and on family and social dynamics. In all instances, qualitative work resonates with clinical work in a patient-centered way that is less abstract than quantitativist research. In brief, it deepens the clinical work, and at its best can be a form of citizen science that involves patients and families in the design and interpretation of the studies, for

All qualitative research in some ways is participatory action research. Both of us are being researched as we speak. You are part of the exchange. We will honor your words as we present them, before we send the paper for publication, we will want to send it to you to make sure that we got things right, or not. That's part of the beauty of qualitative work. *(AM)*

Given its many advantages and the richness of its findings, a common sentiment was still having to justify qualitative methods to others as “real” science, of addressing its existence in medicine (rather than in the humanities). As member of a methodological minority, one participant stated how.

This is what they call a “minority burden.” Always explaining, justifying, “why, how?” Having to tell your story many more times than others do. *(AF)*

On select occasions, some were able to pivot from justifying to teaching: from having their peers ostracize the method to becoming intrigued by it. Some participants went on to implement introductory or advanced courses in qualitative methods, exposing peers to the new epistemology earlier in their professional trajectory. Regardless of its initial specialty focus, the growth of qualitative methods in a medical center or university stands to benefit all disciplines: through incorporation into curricula, research opportunities, availability of mentors, or exposure to different methodologies. In the final analysis, it may well be that.

The job of the quantitative paper is to restore meaning...but meaning runs through all qualitative research because it's based on real encounters and on clinical questions that matter. *(AM)*

## Discussion

Based on the qualitative analysis of individual interviews of CAP clinician-investigators at different stages of professional development and hailing from two different countries, we have identified pathways facilitating or impeding their interest in qualitative research. We organized our overall framework along a temporal sequence, from priming and discovery to transitioning into the field (*Becoming*); through doing, connecting, and belonging (*Being*); and into innovating, refining, mentoring others, and advocating for the discipline (*Nurturing*). We arrange the section that follows along the same three temporal domains, and complemented by the perspectives of the individual investigator (internal factors), the qualitative discipline and the scientific environment (external), and the future development of qualitative research in CAP. Along the way, we emphasize those modifiable factors that may strengthen a quality pipeline: one with more dedicated researchers and greater quality of research.

### *Becoming* a qualitativist: the investigator

We view the transformation of a prospective scientist into a qualitative researcher as subsumed under three categories: grounding, de/centering, and practicalities.

By *grounding* we refer to the personalizing of the investigator’s research focus in such a way that does not jettison prior strengths and interests. To the contrary, investigators, either fledgling or seasoned, can incorporate those priors in a meaningful way. Specifically, previous interest or knowledge in the humanities and social sciences (HSS) provides a template for comfort with uncertainty, concrete tools for textual analysis and deconstruction, and an example for learning from other fields of inquiry.

An emerging literature in medical education, as well as changing practices in medical school admissions committees, suggest the benefits of HSS to medical practice beyond qualitative research. Medical students with HSS premedical education perform on par with peers on more traditional tracks [26]. The Humanities and Medicine Program (HuMed) at the Mount Sinai School of Medicine replicated the finding and found an additional trend toward residency careers in psychiatry and primary care [27]. As an added benefit, humanistic factors taken into account for admission into medical school have been found to promote the selection of physicians with stronger communication skills [28]. Supporting–and indeed encouraging—the application of students from “nontraditional” (i.e. HSS) backgrounds stands to strengthen the medical workforce [29]. A shift in the hidden curriculum (i.e., from scientific exclusivity) toward one of “epistemological inclusion” [29] (i.e. to welcoming HSS) stands to benefit child psychiatry in general, and its qualitative research portfolio in particular.

By *de/centering* we refer to the balance that every qualitative researcher must have between their outer and inner views. At its best, qualitative research relies on “polyocular sampling,” [30] in which multiple viewpoints are incorporated. The outward-facing view—decentering—is particularly relevant in CAP research, where the voices of children need to be incorporated together with those of their caretakers and relevant others. The compelling nature of young lives can be a major research draw, and one that needs to be carefully and ethically balanced by the precondition to conduct research “*with,* rather than *on* children.” [31]. But the inward-facing view—centering—needs to be just as strong. Less experienced investigators may consider this posture self-serving or narcissistic, before coming to realize the centrality of their personal narrative, identity struggles, and overall reflexivity in conducting qualitative work. The degree of inward-facing view can range from minimal hovering (e.g., baseline awareness of relevant conflict), all the way to complete centering on one’s personal experience, as in the case of autoethnography [32].

In contrast to traditional quantitative studies such as randomized control trials, or in brain imaging, epidemiology, or genetics, the *practicalities* of conducting qualitative research are generally facilitators rather than impediments, particularly to new investigators: First, data collection can be completed in a relatively short time (weeks or months, not years); second, given the non-interventional nature of most qualitative studies, institutional review approval usually falls under expedited or exempt categories (whether involving minors or not, respectively); third, synchronized videoconferencing makes interviews and data collection simple, even at geographic remove; fourth, costs are low, additionally so since the advent of AI-supported transcription with platforms such as in Deepgram; finally, analytic methods such as thematic analysis (TA) are accessible and require a modest learning curve–as opposed to grounded theory and other more demanding approaches.

### Being a qualitativist: the discipline and the scientific environment

Medicine and psychiatry at times do not welcome—and often do not understand—qualitative methods or the role they can play in advancing their respective fields. This seeming misalignment in views and scientific priorities can make for a disorienting entry into the qualitative field. Likewise, the lack of common and visible templates and role models, as well of the high flexibility and uncertainty that are inherent to the field [33], all need to be reconciled and overcome. Finding academic lodging within groups and institutions that espouse scientific openness is one way to do so: *epistemological flexibility*—the ability to use the right methodology for a given task, as opposed to embracing preconceived scientific notions—is not only to be sought and embraced, but indeed developed and fostered by maturing investigators and educators.

The principles of narrative medicine (NM) [34] can provide a useful bridge, particularly at a time of high patient unease and physician discontent: “Clinical practice fortified by *narrative competence*—the capacity to recognize, absorb, metabolize, interpret, and be moved by stories of illness” [35]—can help close the gaps between patients and providers. In the case of psychiatry, it can help close its gap with medicine; to bring mind and body closer in line. Qualitative methods can further and complement the goals of NM through scholarly work and research. Like NM, qualitative methods—beginning with their interviews and focus groups—harness the fact that we are storytelling beings eager to tell our own stories and listen to the ones of those we are interested in learning from—and with. The revitalizing force of storytelling in psychiatry is far from new and has continued to change organically with the technologies of the times, as exemplified by the Multimedia Digital Storytelling in Psychiatry project [36].

Albert Einstein is credited with saying that “not everything that can be counted counts, and not everything that counts can be counted.” The possibly apocryphal line is relevant in the context of qualitative research in medicine and psychiatry, areas in which citation numbers and impact factors are consistently low for qualitative when compared to quantitative science. Despite optimistic prognostications [37] of greater acceptance and assimilation of qualitative methods into the “medical model,” the divisive current state of publication affairs is unlikely to change anytime soon, as journals compete for higher ratings, and a wider array of alternative outlets become available. Stated differently: a coming together under the banner of epistemological flexibility remains aspirational. Submissions will continue to go to separate and more specialized journals, with only a few periodicals able to straddle the field (or interested in doing so). Once again, the narrative perspective (like Einstein's words) may provide an important salve: “Before psychiatry rushes in to ‘save’ its bioscientific self, however, it seems this moment offers an opportunity for self-reflection and deeper understanding of the process of psychiatric meaning-making…The implication of narrative for psychiatry is that there are many ways to tell the story of mental health problems—not just one right way and many other wrong ways [38].”

Among the challenges identified toward the integration of qualitative methods into the medical scientific mainstream is the fact that journals and funders commonly use evaluation criteria that are incongruent with qualitative methods and constructivist epistemology. Ungar [30] offered four different proposals to address this chasm through, and which can be construed either as creative solutions or as self-defeating concessions: (i)* Dressing up,* in which a qualitativist is incorporated into a larger project (and funded by it), but where their contribution is seen as supplemental to the “real work” under way, invariably culminating with the quantitative analysis of a large-sample dataset; (ii)* Sleeping with the elephant,* which involves a practical path to funding by creating mixed-method designs that leave all parties satisfied. The challenge in mixed-method designs is to reach a detente between researchers from different paradigms; (iii)* Seek but never find:* accepting one’s role as a qualitativist, but only en route to a “real” yet at times elusive (quantitative) study; and (iv) *Table scraps.* Be satisfied with small funding requests, especially if aligned with service delivery rather than research. As noted above, table scraps may be sufficient to support a qualitative research project. Universities and foundations are commonly able to fund seed grants of smaller dollar amount; however, the longer-term challenge is that they will rarely provide salary support (Table 4).

**Table 4:** Strategies to enhance entry into qualitative careers and research in child and adolescent psychiatry

Level of intervention	Category	Potential strategies
*Becoming *a qualitativist: the investigator (internal factors)	*Grounding:* personalizing the investigator’s research focus	• Previous interest or knowledge in the humanities and social sciences can provide a useful template for qualitative research • A shift in the hidden curriculum (i.e., from scientific exclusivity) toward one of “epistemological inclusion” (i.e., to welcoming humanities and social sciences) stands to benefit qualitative researchers
	*De/centering:* balancing inward and outward views	• Foster “polyocular sampling,” in which multiple viewpoints are incorporated, including: • Outward-facing views—decentering—through which to incorporate the voices of children, their caretakers, and relevant others • Inward-facing views—centering—through which to bring reflexivity and oneself into the work
	*Practicalities:* leveraging advantages	• Data collection can be completed in a weeks or months, not years • Institutional review approval usually falls under expedited or exempt categories • Synchronized videoconferencing makes interviews and data collection simple, even at geographic remove • Costs are low, additionally so since the advent of AI-supported transcription • Analytic methods such as thematic analysis (TA) are accessible and require a modest learning curve
*Being* a qualitativist: the discipline and the scientific environment (external factors)	*Epistemological flexibility*	• Finding academic lodging within groups and institutions that espouse scientific openness can be conducive to success as a qualitativist • Narrative competence—the capacity to recognize, absorb, metabolize, interpret, and be moved by stories of illness”—can help close gaps between patients and providers
	*Recounting:* moving beyond bibliometrics	• “Not everything that can be counted counts, and not everything that counts can be counted” (attributed to Einstein) • There are many ways to tell the story of mental health problems—not just one right way and many wrong ways
	*Dis/integrating:* mixing and unmixing methods	• Joining a mixed methods project as a qualitativist has natural appeal and fosters collaboration across epistemologies • There are risks to be considered as well, including being tokenized or relegated to an irrelevant role
*Nurturing* quality: the future	*Capacity building:* invigorating a quality pipeline	• Organized teaching of alternative approaches to science, such as qualitative methods within a medical context • Making use of a broadening literature and expertise on incorporating qualitative methods into medical education
	*Joining:* building communities	• Education is necessary but not sufficient in pursuit of qualitative competence; theory and learning need to come alive in practice • Communities of practice can be a prime way to launch into a first study accompanied, feeling support and guidance at such a critical career juncture

### *Nurturing* quality: the future

In our view, the longer-term success of qualitativists in CAP—their movement from pathways into identity—hinges on two main factors: *Capacity building,* the invigoration of a quality pipeline through education and early exposure; and *Joining,* the strengthening and commitment through an enduring community.

With respect to education, one of our study participants wondered why it is that the word “epistemology” is never used in the context of learning about quantitative methods: “Isn’t an objectivist epistemology just as important as a constructivist one?” The observation was telling: Qualitative methods (and their underlying epistemology) are usually defined not by themselves, but rather in contrast to quantitative methods and their underlying epistemology. These observations, which usually animate first introductory lessons, invite the question over the optimal way, timing, and curricular placement of how and when best to introduce qualitative methodology into medical and psychiatric education. As noted, qualitative methodologies are often at odds with the objectivist epistemology deeply embedded in medical school settings. The success of a qualitativist in medicine will depend on being knowledgeable about their methods, but also of being “better prepared to successfully negotiate the politics of science, the politics of evidence, and the politics of funding within their home institutions [39].”

One way of addressing the politics of “methodological conservativism” [40] is through the deliberate and organized teaching of alternative approaches to “traditional” science, such as qualitative methods within a medical context. In addition to foundational concepts such as sampling, questionnaire development, or data collection and analysis, some qualitative concepts may be hard to grasp for someone socialized under traditional medical mores. As such, additional emphasis on topics such as reflexivity (as opposed to bias), or transferability (as opposed to external validity), will be important pedagogic investments to any successful course addressing qualitative methods within medicine [41]. There is by now a broadening literature and expertise on incorporating qualitative methods into medical education [42], and specifically into mental health, where they remain underutilized [37]. Adaptations to medical science include important lessons from nursing science as well. For example, interpretive description (ID) is a qualitative approach first developed by nursing that has deliberately practical, applied, here-and-now goals [43]. Aimed at circulating research findings quickly back “to the bedside,” ID incorporates quality improvement and practical aspects at a timescale and applicability relevant to medicine and nursing in ways very different from those of sociology or anthropology, examples of two foundational sciences behind qualitative methodology.

A community of practice (CoP) is a group of like-minded individuals who share a specific interest or area of expertise. First described outside of the realm of medicine [44], the CoP construct has proven fruitful in it [45, 46]. Medical CoPs have been organized around particular specialties, emerging areas of interest, or the refining of evolving technical skills. CoPs have the added function of bringing together different cohorts, which in turn helps with the intergenerational transmission, refinement, and preservation of skills and knowledge. CoPs provide an entry point for novice learners—being more welcoming, personalized, and less diffuse and overwhelming than large society meetings tend to be.

CoPs can be a prime way to launch into a first study accompanied, feeling support and guidance at such a critical career juncture. Education is necessary but not sufficient in pursuit of qualitative competence; Theory and learning need to come alive in practice. Members of a CoP may propose ideas, or invite a new member to participate in an ongoing project. In that spirit, we formed a qualitative CoP in 2022. Through the binational partnership between the Yale Child Study Center in the US, and the Centre de Recherche en Épidémiologie et Santé des Populations in France, we developed in 2022 Qua*Lab*, the Qualitative and Mixed Methods Lab. Our group has since grown to include members from Canada, Brazil, and the Dominican Republic. As a group, we meet twice monthly to review protocols and manuscripts under preparation. We welcome, and indeed encourage the participation of medical students along senior faculty, and are committed to the growth and generational transmission of qualitative methods. One way of fomenting such growth is through peer-near support, in which junior participants guide more recent or inexperienced members. Critically, as young mentors, they are in turn provided with senior support to assuages worries and prevent them from feeling “farmed out,” or of carrying others on their (junior) backs. In this way, our effort has resulted in a virtual cycle of qualitative development [47–55]. We hope others will join us and create their own CoPs to support quality growth in CAP.

### Limitations

We acknowledge several limitations. First, we did not interview CAP researchers with a predominant or exclusive quantitative focus. Such extreme sampling could have been informative and “kept us honest” regarding our conclusions about their research and their world views. We would also have learned about their perceptions, as “outsiders,” of qualitative work. Second, we recognize than in a polarized view of epistemologies, we failed to incorporate mixed methods research in a meaningful way. Several of the authors have conducted and published mixed methods research; some of them mentioned it during their interviews. However, the approach was difficult to isolate for analysis in this study; it may become a fruitful subject for future research. Third, through an exclusive focus on CAP clinician-investigators, we missed insights that others could provide, starting with psychiatrists, pediatricians, and physicians more broadly, as well as social workers, nurses, psychologists, and other allied professionals.

A final and noteworthy omission is worth pointing out: During their interviews, not a single one of the study participants mentioned large language models (LLMs, such as ChatGPT) and the disruption they are sure to bring into qualitative methodologies [56, 57]. The omission could be related to a lack of knowledge (e.g., about LLMs), to a failure of imagination (e.g., of their possible applications), or perhaps even to an existential threat (e.g., “will we become the tools of our tools?”). It is clear that we are at the dawn of a methodological revolution: in an exponential way, LLMs will save time, reduce costs, and increase the throughput of coded materials. But gathering rich data through interviews, and planning and interpreting results will still require emotionally competent human researchers. LLM-assisted qualitative research may become faster and stronger, but the accuracy, novelty, and relevance of its results will still depend on humans: We shall not become obsolete.

### Supplementary Information

**Additional file 1.** INTERVIEW GUIDE - Sensitizing questions.

## Data Availability

Supporting data will be provided by the corresponding author upon reasonable request.
