# Supplementary material for: Pathways and identity: toward qualitative research careers in child and adolescent psychiatry
Source: Child Adolesc Psychiatry Ment Health. 2024 Apr 29;18:49. doi: 10.1186/s13034-024-00738-8 (PMC11059710; doi:10.1186/s13034-024-00738-8)
Supplement: Supplementary file 1 — Additional file 1. INTERVIEW GUIDE - Sensitizing questions. [file 13034_2024_738_MOESM1_ESM.docx]

**APPENDIX S1**

**INTERVIEW GUIDE - Sensitizing questions**

1. What do you like about qualitative research?
2. When did you first learn about qual research?
3. How did you come to qual research?
   1. who was involved in supporting you? [Support]
   2. was someone preventing you from entering this field? [Barrier]
4. What topic did you choose at the beginning? The first topic that resonated with you
5. Who does qualitative research with you [solitude/togetherness]?
6. How was your research perceived by people who can influence your career?
7. How did becoming a qualitative researcher impact your professional life as a clinician? [Transformational process: reflexivity, constructivism]
8. Has your qualitative practice changed over time? [expertise; different methodologies - flexibility or rigidity towards using different methods - how are methods perceived by others: phenomenology too close to philosophy?]
9. What is specific about qualitative research in Child and Adolescent Psychiatry? [clinical work, effect of interviews on patients, developmental aspect]

[which elements are specific to pediatrics, and which are more specific to psychiatry? and the intersection of both?]

1. What has been exciting about qual research versus disappointing?
2. What about QUANT research: what has been exciting versus disappointing with QUANT?
3. Think about your favorite qualitative paper. What does it mean to you?
4. Think about your favorite quantitative paper. What does it mean to you?
5. How does qual research impact your funding strategy/opportunities?
6. How does QUAL research impact promotion opportunities? How do you navigate that?
7. How does your identity as a qualitativist intersects with your identity as Child and Adolescent Psychiatry? Or … What does your qualitative research brings to your work as CAP?
8. How is your qual research perceived by non-qualitativist CAPs (reviewers, audience in conferences)?
9. Junior QUAL: how does the fact of having published mainly qualitative papers impact your residency application strategy?
10. Junior QUAL: what was your background before medical school? [US: undergrad]
11. Junior QUAL: how is qualitative research appraised/evaluated compared to quantitative research among your peers?
12. Junior QUAL: Describe your experience finding a qualitative research mentor(s)
13. Junior QUAL: What is the tone surrounding qualitative research in medical school? Is it an option that is discussed? Are students encouraged to explore alternative research methodologies outside the typical clinical/quant work? Please elaborate.
14. What would it look like if you could wave a magic wand and imagine a perfect world of child and adolescent psychiatry research?
